# Study on the Regulation of Early-Age Deformation Characteristics of High-Strength Coral Sand Grouting Materials

**DOI:** 10.3390/ma18163740

**Published:** 2025-08-10

**Authors:** Dongxu Hou, Wei Li, Xiao Xue, Zhi Liu, Hongwei Han, Yudong Han

**Affiliations:** 1 School of Water Conservancy and Civil Engineering, Northeast Agricultural University, Harbin 150030, China; 18346238209@163.com (D.H.); 17691007992@163.com (X.X.); 17853819179@163.com (Z.L.); 2 Central Research Institute of Building and Construction Co., Ltd., MCC Group, Beijing 100088, China; mcc_liwei@163.com; 3 Heilongjiang Provincial Key Laboratory of Water Resources and Water Conservancy Engineering in Cold Region, Northeast Agricultural University, Harbin 150030, China

**Keywords:** coral sand grouting materials, shrinkage deformation, early strength agent, combination regulation, sealed and dry curing conditions

## Abstract

Coral sand grouting materials can effectively meet the new development requirements of remote island and reef engineering projects, demonstrating significant application value. However, its early-age shrinkage deformation may compromise structural stability. To effectively regulate this early shrinkage behavior, this study investigated the influence of varying dosages of early strength agent (ES), plastic expansive agent (PEA), and post-hardening expansive agent (HP-CSA) on the complete vertical expansion rate curve of coral sand grouting materials during 0–48 h, while comparatively examining the combined effects of composite expansive agents on early autogenous shrinkage and drying shrinkage characteristics. The results show that during 0–48 h, ES and composite expansive agents can precisely control the activation window of PEA, enabling controllable development of ultra-early vertical expansion in the grouting material, with increased HP-CSA dosage accelerating the progression of the complete vertical expansion rate curve. From 2 to 28 days, the coral sand grouting materials exhibit continuous shrinkage development. An appropriate combination of PEA and HP-CSA effectively synergizes to regulate shrinkage deformation. The drying shrinkage significantly correlates with the water loss rate. Within the scope of this study, when the dosages of ES, PEA, and HP-CSA are 1%, 0.06%, and 4%, respectively, the performance of coral sand grouting materials is relatively good.

## 1. Introduction

With the advancing development of marine resources, offshore island and reef engineering construction has grown rapidly [1,2]. Coral reefs, as unique natural resources in these regions, contain over 96% CaCO_3_. After the death of coral organisms, they undergo long-term deposition and weathering to form coral aggregates. These aggregates can be locally utilized to replace conventional aggregates in concrete production [3,4,5], alleviating the scarcity of high-quality aggregates on islands and the high transportation costs associated with traditional sand and stone aggregates [6,7,8]. Da et al. [9] performed a comparative analysis of stress–strain curves between coral concrete and ordinary concrete at equivalent strength levels, revealing that the descending period of the coral concrete curve is steeper than that of ordinary concrete, indicating higher brittleness in coral concrete at comparable strength. Wang et al. [10] prepared C20–C40 coral concrete using coral aggregates with two particle size ranges of 5.0–25.0 mm and 0.15–4.75 mm and seawater. Microscopic tests showed that coral aggregates form stronger bonds with conventional aggregates and exhibit denser interfacial transition zones than conventional aggregates. Zhou et al. [11] produced coral concrete by gradually replacing limestone aggregates with coral coarse aggregates larger than 4.75 mm, finding that when the coral aggregate content does not exceed 20%, the specimens achieve 28-day compressive strengths exceeding 60 MPa. Cheng et al. [12,13] investigated the effects of combined mineral admixtures on the compressive strength and durability of coral aggregate concrete. The coral aggregates used had a maximum particle size of 4.75 mm and a fineness modulus of 2.27. The finally prepared coral aggregate concrete’s 28-day compressive strength was 47.8 MPa.

Currently, offshore island and reef engineering construction is advancing toward high-quality and high-performance development, focusing on performance enhancement and regular maintenance of existing structures [14,15]. Cement-based grouting materials, characterized by high fluidity, slight expansion, and superior strength, are widely applied in structural repair, building reinforcement, and engineering protection [16,17,18], thus effectively meeting the requirements of the new development stage of island and reef construction. Traditional grouting materials predominantly utilize silica sand or river sand as aggregates [19,20]. Li et al. [21] used ISO standard sand (the main component is silica sand [22]) to investigate the mechanism of superabsorbent polymers as curing agents in grouting materials, revealing their capacity to absorb excess water during mixing and subsequently release it for internal curing, thereby improving strength. Burnwal et al. [23] developed grouting materials with river sand as fine aggregate, which achieved a 28-day compressive strength of 41.5 MPa under room-temperature curing. Few studies have been conducted on developing novel grouting materials using coral aggregates, considering the resource availability and practical demands of island or reef regions.

Grouting materials undergo shrinkage deformation during setting and hardening, which significantly impairs structural integrity and long-term engineering stability, making effective control of such shrinkage crucial [24,25]. Current national standards [26] primarily focus on ultra-early-age deformation control, specifying two key parameters: the 3 h vertical expansion rate and the difference between the 24 h and 3 h vertical expansion rates. Traditional grouting materials rely mainly on PEA to achieve ultra-early expansion, with azo-compound-based agents being the most prevalent. These generate physical expansion by releasing nitrogen and other gases through reactions in an alkaline environment. However, if grouting materials exhibit excessive fluidity or delayed setting, the generated gases will escape without forming effective expansion. Conversely, if early strength develops too rapidly with increased restraint, the trapped gases within the matrix fail to create sufficient expansion [27,28]. Furthermore, the vertical expansion induced by PEA typically terminates within 24 h, whereas shrinkage deformation develops rapidly in subsequent stages. A multitude of researchers have investigated various types of expansive agents, offering diverse approaches for deformation control in grouting materials [29,30]. Zhang et al. [31] investigated the effects of varying dosages of PEA on the performance of grouting materials, revealing that an increased dosage enhances the vertical expansion rate but subsequently elevates drying shrinkage. Carballosa et al. [32] compared calcium oxide additive and calcium sulfoaluminate additive, demonstrating that the calcium oxide type exhibits superior expansion performance and stability. Hu et al. [33] examined the microstructural pore evolution at different ages, finding that calcium sulfoaluminate expansive agent accelerates hydration and promotes the formation of ettringite and C-S-H gels.

Additionally, properly incorporating ES can promote early hydration development and enhance the initial strength of grouting materials [34,35,36]. Du et al. [37] systematically studied the influence of lithium carbonate dosage on the compressive strength of grouting materials, showing that the compressive strength first increases and then decreases with increasing dosage, with an optimal dosage of 0.02% (relative to the mass of cement). Chen et al. [38] investigated the mechanisms by which different types of ES affect the early hydration process of grouting materials and developed a composite ES using calcium formate and sodium sulfate. With appropriate incorporation, the 3 h compressive strength of grouting materials can reach 16.7 MPa. Currently, research on the regulatory design of early-age shrinkage deformation in grouting materials using ES and composite expansive agents remains relatively limited. Even fewer studies focus on controlling the early shrinkage deformation characteristics of coral sand grouting materials, a novel type of material.

Based on the above analysis, this study conducted experiments on the complete 0–48 h vertical expansion curves of coral sand grouting materials with varying dosages of early strength, plastic, and hardened expansive agents. Furthermore, we determined the optimal dosage of ES based on the Class II fluidity and vertical expansion rate requirements specified in the current national standard for grouting materials and investigated the effects of composite expansive agents on the patterns of autogenous shrinkage and drying shrinkage over 2–28 days. The specific standards are shown in Table 1. The results enable controllable regulation of ultra-early-age vertical expansion in coral sand grouting materials and provide data support for controlling early-age shrinkage deformation.

## 2. Materials and Methods

### 2.1. Raw Materials

**Figure 1:**
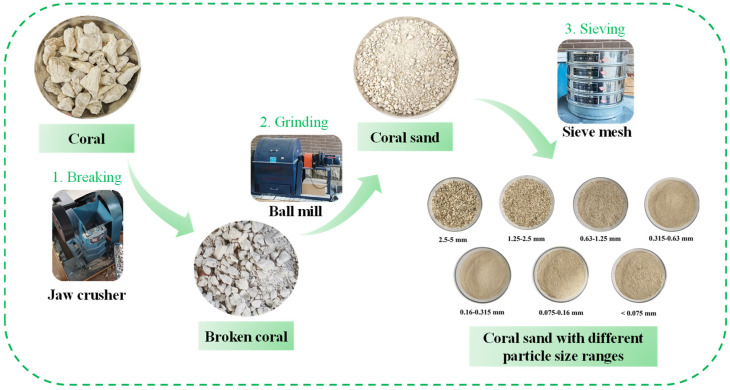
The processing technology of coral sand.

**Figure 2:**
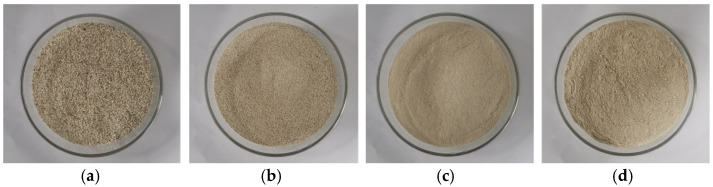
Preferred fine coral sand with different particle size ranges: (**a**) 0.63–1.25 mm; (**b**) 0.315–0.63 mm; (**c**) 0.16–0.315 mm; (**d**) 0.075–0.16 mm.

The cement used in this study was P·II 52.5R Portland cement produced in Nanjing. The mineral admixture was fly ash microspheres (FFA) from Tangshan, Hebei. The HP-CSA was calcium sulfoaluminate–calcium oxide expansive agent produced in Tianjin, with its mineral composition shown in Table 2. The water reducer (PS) was a polycarboxylate superplasticizer from Shanghai with a ≥ 21% water reduction rate, powder form. The PEA, defoamer (DA), and redispersible polymer powder (RPP) were all produced in Shanghai. The ES was calcium formate from Tianjin. We collected coral aggregates from an offshore construction site and processed the coral aggregates through crushing, grinding, and sieving to obtain optimally graded coral sand with different particle size ranges. The final coral sand product features a maximum particle size of 1.25 mm and a fineness modulus of 2.1. The preparation process and results of coral sand are shown in Figure 1 and Figure 2.

### 2.2. Mix Proportion Design

Based on the previously conducted mix proportion optimization tests [39], the mix proportions of the coral sand grouting materials were determined as shown in Table 3, with a water-to-binder ratio of 0.24, binder-to-sand ratio of 1.0, and FFA dosage of 30% (by mass of the cementitious materials, the same below). On this basis, the experiment used ES, PEA, and HP-CSA as variables, designing ES (external addition) dosages of 0%, 1%, and 2%, PEA (external addition) dosages of 0.04%, 0.06%, and 0.08%, and HP-CSA (internal addition) dosages of 2% and 4%, labeled as ES0P4H2 to ES2P8H4, totaling 18 mix proportions, as shown in Table 4. We investigated their effects on the fluidity, mechanical properties, and 0–48 h vertical expansion rate curve of coral sand grouting materials. Furthermore, with the optimal ES dosage fixed, we investigated the influence of the composite expansive agents on the shrinkage deformation of coral sand grouting materials under sealed and dry curing conditions from 2 to 28 days.

### 2.3. Experimental Methods

**Figure 3:**
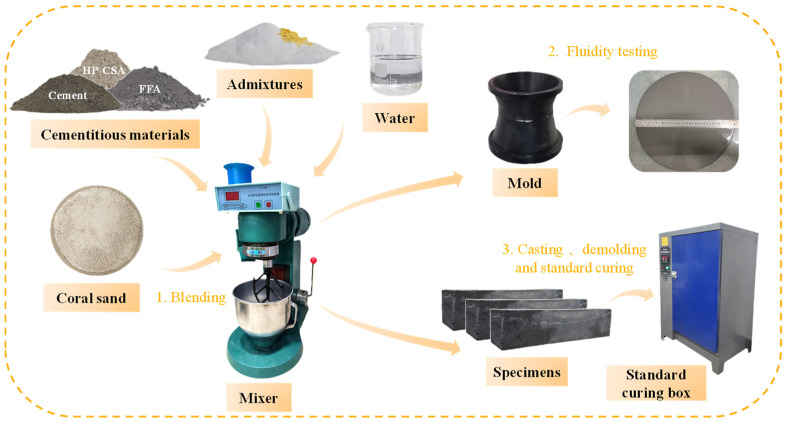
The preparation process of coral sand grouting materials.

Figure 3 shows the preparation process of coral sand grouting materials. First, coral sand and cementitious materials were loaded into a mixer and blended for 1 min. Then, admixtures were added and mixed for 30 s. After thorough homogenization, the mixing water was introduced and stirred for approximately 3–4 min to obtain the final coral sand grouting materials. The resulting slurry was immediately poured into a truncated cone mold for fluidity testing, while the remaining portion was cast into compressive strength molds for subsequent curing and demolding.

**Figure 4:**
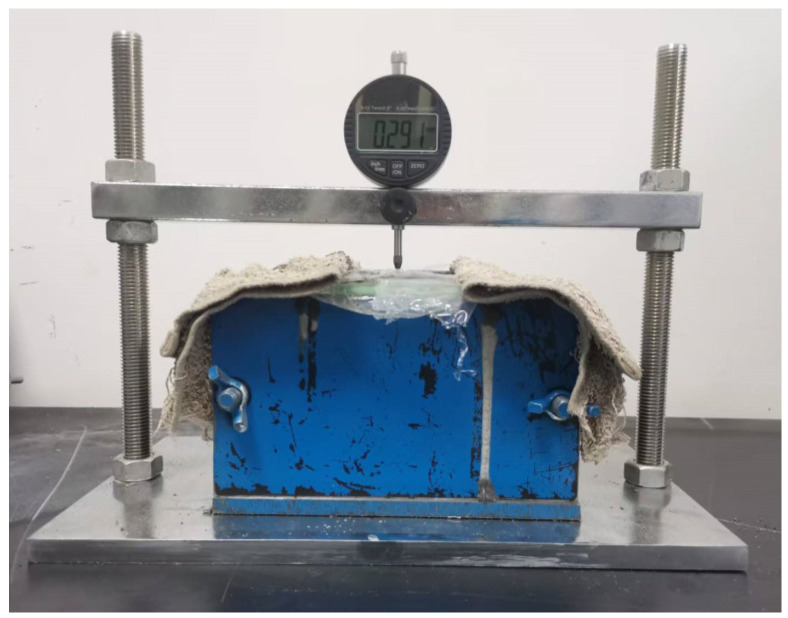
Vertical expansion rate tester.

**Figure 5:**
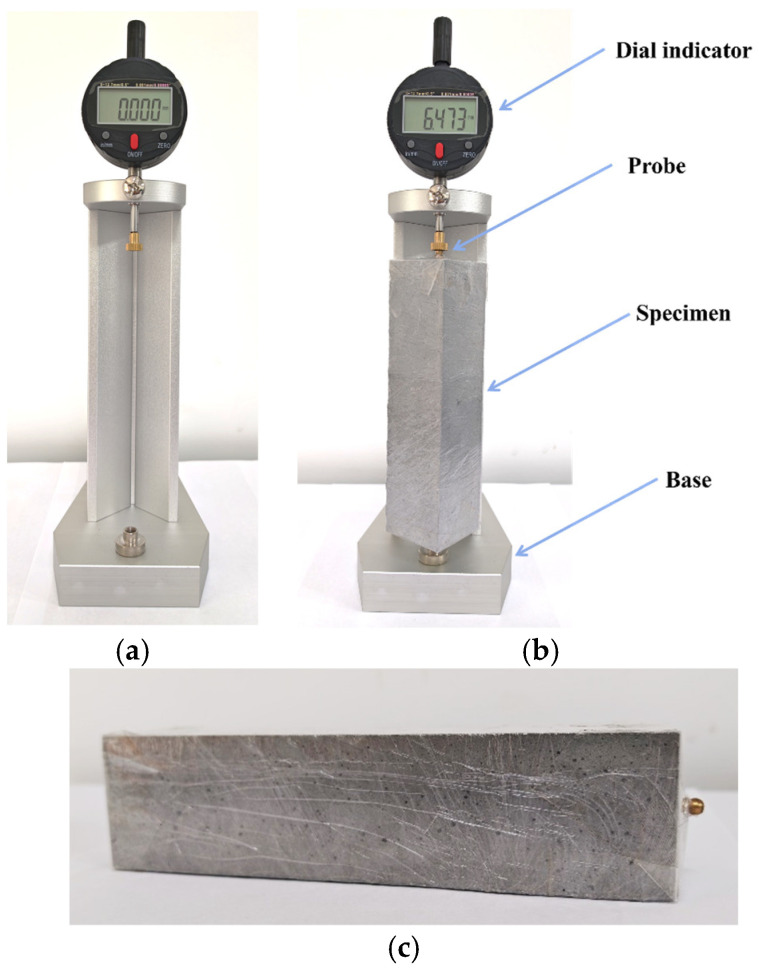
Test instrument and specimen preparation: (**a**,**b**) early shrinkage performance test instrument; (**c**) sealed specimen.

The initial fluidity and 30 min fluidity of coral sand grouting materials were tested according to Appendix A.0.2 of GB/T 50448-2015 “Technical Code for Application of Cementitious Grout” [26]. The vertical expansion rate within 48 h was measured following Appendix A.0.6 under laboratory conditions of (20 ± 2) °C and (60 ± 5) % RH, using the test instrument shown in Figure 4. For the initial 24 h period, we recorded expansion measurements at 30 min intervals, which were reduced to hourly readings from 24 to 48 h. The shrinkage deformation from 2 to 28 days was tested by JGJ/T 70-2009 “Standard for Test Method of Performance on Building Mortar” [40], using the test instrument shown in Figure 5a,b. At 2 days of age, the specimens were demolded and subjected to sealed or dry curing conditions, with shrinkage values recorded daily. The sealed specimens were tightly wrapped with industrial plastic film as illustrated in Figure 5c. Additionally, to characterize water loss under dry curing conditions, the mass change in specimens was concurrently recorded, with the drying water loss rate (water loss mass per unit volume) serving as the evaluation index [41], and calculated using Equation (1). The compressive strength was tested according to GB/T 17671-2021 “Test Method of Cement Mortar Strength (ISO Method)” [22], with specimens demolded at 1 day of age and transferred to a standard curing box until testing at specified ages.

(1) $$w_{L}=\frac{m_{w}}{V}$$

where $w_{L}$ is drying water loss rate, $m_{w}$ is the weight of lost water, and V is the volume of coral sand grouting materials.

## 3. Results and Discussion

### 3.1. The Development of the Vertical Expansion Rate from 0 to 48 Hours

**Figure 6:**
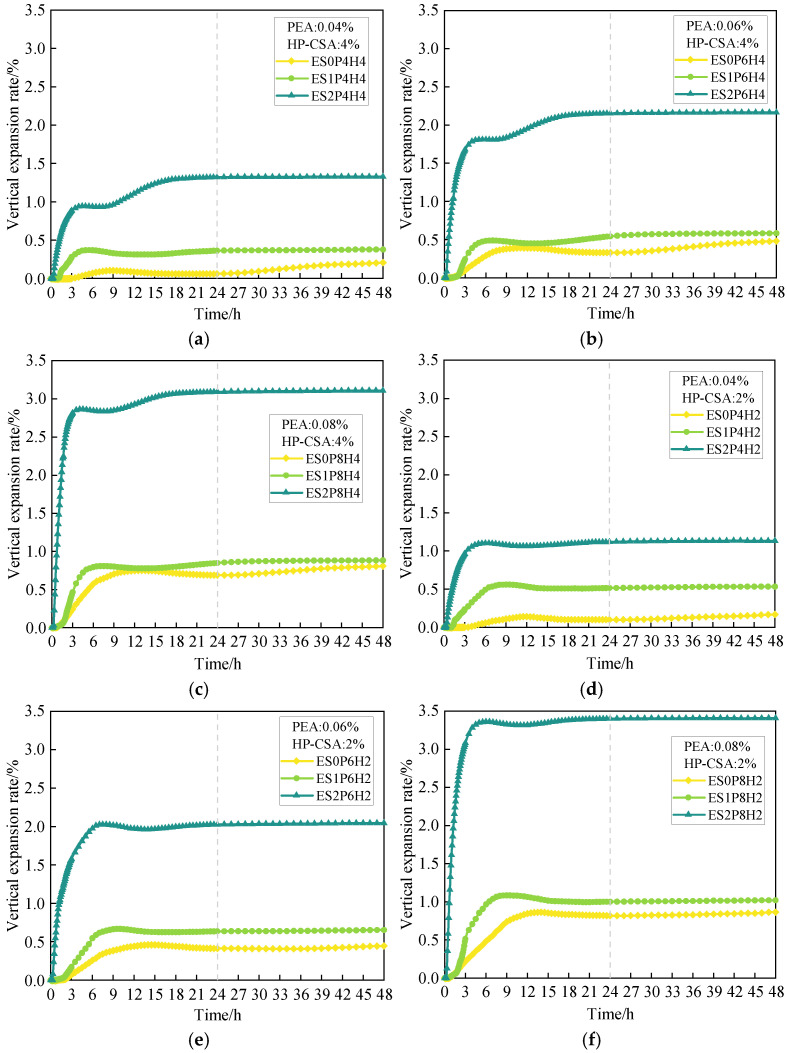
The effect of different dosages of “ES + PEA + HP-CSA” on the vertical expansion rate of coral sand grouting materials within 48 h: (**a**) PEA: 0.04%, HP-CSA: 4%; (**b**) PEA: 0.06%, HP-CSA: 4%; (**c**) PEA: 0.08%, HP-CSA: 4%; (**d**) PEA: 0.04%, HP-CSA: 2%; (**e**) PEA: 0.06%, HP-CSA: 2%; (**f**) PEA: 0.08%, HP-CSA: 2%.

Figure 6 shows the complete vertical expansion curves of coral sand grouting materials, incorporating varying dosages of ES and composite expansive agents within 48 h. As shown in Figure 6a–c, when the dosage of HP-CSA is 4%, with a constant dosage of PEA, the incorporation of ES can significantly increase the vertical expansion rate of coral sand grouting materials within 3 h. To illustrate, for the three test groups with a PEA dosage of 0.06%, when the ES dosages are 0, 1% and 2%, the 3 h vertical expansion rates of coral sand grouting materials are 0.097%, 0.247%, and 1.681%, respectively. The phenomenon arises because ES promotes ultra-early matrix strength development, mitigates the retarding effect in coral sand grouting materials, and aligns strength development with PEA’s gas generation window, effectively preventing early gas escape and thereby maximizing PEA’s expansive effect within 3 h. Moreover, the synergistic effect between ES and PEA on 3 h expansion becomes more pronounced with higher PEA dosage. For ES2P4H4, the 3 h vertical expansion rate increases by 0.885% relative to the ES-free counterpart within the same series. Similarly, when the PEA dosage increases from 0.06% to 0.08%, these increments rise to 1.584% and 2.325%, respectively, demonstrating a nonlinear amplification exceeding the PEA dosage increase.

During 3–24 h, all groups satisfy the national standard requirements for the difference in vertical expansion rates. At 3 h, when PEA is still in its rapid reaction phase, the incorporation of ES both enhances gas retention from PEA and significantly advances the peak time of the vertical expansion curve in coral sand grouting materials. Higher ES dosages exhibit a more substantial inhibitory effect on retardation, thereby accelerating the cement hydration rate and the temperature rise in grouting materials, which in turn advanced the thermal expansion–contraction deformation process in the slurry.

After peaking, the vertical expansion curve enters a slight declining phase, which corresponds to the post-peak deceleration stage of the cement hydration reaction rate [42]. During this period, the expansive capacity of PEA in coral sand grouting materials has diminished notably, while the rate of chemical shrinkage induced by cement hydration remains at its peak. Meanwhile, the decrease in hydration temperature induces cooling-driven shrinkage of the system, and the bubbles formed in the early stage within the paste destabilize and rupture due to variations in environmental conditions. These factors collectively result in a decline in the vertical expansion rate curve of the coral sand grouting materials. Subsequently, as the cement hydration rate gradually decreases, chemical shrinkage diminishes, and the specimen temperature stabilizes to ambient levels; the cooling contraction process ceases. At this point, although the rate of matrix autogenous shrinkage decreases as hydration slows, it continues to progress. Meanwhile, the expansive products generated by HP-CSA hydration accumulate progressively, inducing slight expansion. These combined effects convert the initial brief decline into a decelerating micro-expansion, which continues until equilibrium is reached at 48 h.

During 24–48 h, the expansive effect of PEA ceases, and HP-CSA becomes the dominant factor governing the post-hardening vertical expansion of coral sand grouting materials. At a constant PEA dosage, increasing ES dosage enhances the compressive strength at 1 d, thereby strengthening the matrix’s restraint on the expansive performance of HP-CSA and inhibiting sustained micro-expansion. With the PEA dosage fixed at 0.04%, when the ES dosages are 1% and 2%, the differences in vertical expansion rates between 24 and 48 h are 0.015% and 0.008%, which are 89.7% and 94.5% lower relative to the ES-free group, respectively. For PEA dosage of 0.08%, the decreases are 72.0% and 85.6%. When ES dosage reaches 2%, coral sand grouting materials lose capacity for significant post-hardening micro-expansion during 24–48 h.

As shown in Figure 6d–f, when the HP-CSA dosage is reduced to 2%, the overall development trend of the 48 h vertical expansion curves for coral sand grouting materials remains similar to that observed with 4% HP-CSA. It is found that all groups except ES0P4H2, ES0P6H2, and ES0P8H2 meet the national standard requirements. Meanwhile, in all groups with 2% HP-CSA, the first peak values of vertical expansion rates exceed those in the 4% HP-CSA groups, with the corresponding times to reach the first peak being prolonged. For example, the first peak value of ES1P6H2 is 36.1% higher than that of ES1P6H4. This phenomenon arises because HP-CSA, primarily composed of CaO, exerts a degree of setting acceleration effect, enhancing the matrix’s early-age strength to some extent [43,44], thereby hastening the transition to the plastic and hardened stages and ultimately advancing the overall vertical expansion process.

Furthermore, after incorporating ES, in all groups with 4% HP-CSA, the secondary growth magnitudes of vertical expansion rates during the transition from the stage of increasing deceleration to the final equilibrium state are higher than those in all corresponding groups with 2% HP-CSA. A comparative analysis of the differences between the vertical expansion rates at 48 h and those at the lowest points of the descending segments reveals that, for instance, the values for ES1P6H4, ES2P6H4, ES1P6H2, and ES2P6H2 are 0.131%, 0.359%, 0.028%, and 0.084%, respectively. This phenomenon may arise from the acceleration of matrix hydration by ES, which indirectly promotes the reaction of HP-CSA to form expansive ettringite and calcium hydroxide crystals, with volumes larger than those of the original components [45,46]. Additionally, ES used in this study is calcium formate, which exhibits weak acidity in water. It promotes the dissolution of gypsum and the hydration of tricalcium silicate to a certain extent and accelerates the formation of reactants such as ettringite and calcium hydroxide crystals. [38,47,48]. As the matrix has not yet fully hardened at this stage and practical constraints have not yet fully developed, ettringite and calcium hydroxide crystals, within the confined space, induce effective expansive deformation in the material. Under the combined action of the aforementioned factors, the vertical expansion rate of the grouting material exhibits a secondary increase; as the dosage of both increases, the reaction becomes more intense and the increment in vertical expansion rate gradually enlarges, yet as the matrix strength increases, the rate gradually slows.

Notably, when the dosage of HP-CSA is 4% and ES is 1% or 2%, grouting materials can eventually reach a vertical expansion rate higher than its first peak point after going through the deceleration rate growth stage. When the dosage of HP-CSA is 2% and only the dosage of ES is 2%, the above effect is satisfied, but the expansion effect is insignificant. Thus, this study reasonably infers that, under the condition of ensuring the presence of effective free water in the system (to meet the requirements of the HP-CSA reaction as much as possible), incorporating an appropriate dosage of HP-CSA and calcium formate can effectively trigger the secondary increase in the vertical expansion rate of high-fluidity coral sand grouting materials at an ultra-early age.

### 3.2. Fluidity and Mechanical Strength

**Figure 7:**
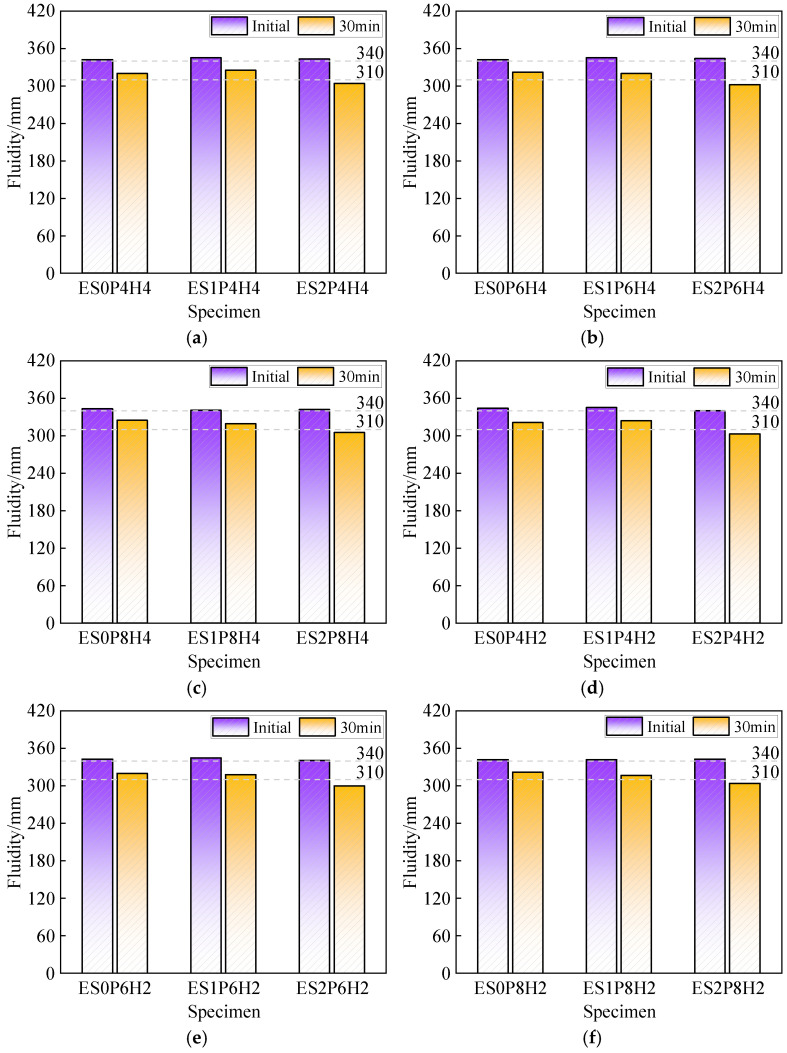
The effect of different dosages of “ES + PEA + HP-CSA” on the fluidity of coral sand grouting materials: (**a**) PEA: 0.04%, HP-CSA: 4%; (**b**) PEA: 0.06%, HP-CSA: 4%; (**c**) PEA: 0.08%, HP-CSA: 4%; (**d**) PEA: 0.04%, HP-CSA: 2%; (**e**) PEA: 0.06%, HP-CSA: 2%; (**f**) PEA: 0.08%, HP-CSA: 2%.

Figure 7 illustrates the effects of varying dosages of ES and composite expansive agents on the fluidity of coral sand grouting materials. Composite expansive agents exhibit a negligible influence on fluidity at a constant ES dosage. With fixed composite expansive agent dosage, 1% ES exerts no significant influence on fluidity, whereas 2% ES reduces the 30 min fluidity retention to below the specified minimum values of 310 mm. The phenomenon arises because ES accelerates the slurry’s hydration reactions, shortening the setting and hardening time. Higher ES dosages further reduce this duration, ultimately resulting in grouting materials’ 30 min fluidity retention failing to satisfy the Class II fluidity requirements specified in the national standard.

**Figure 8:**
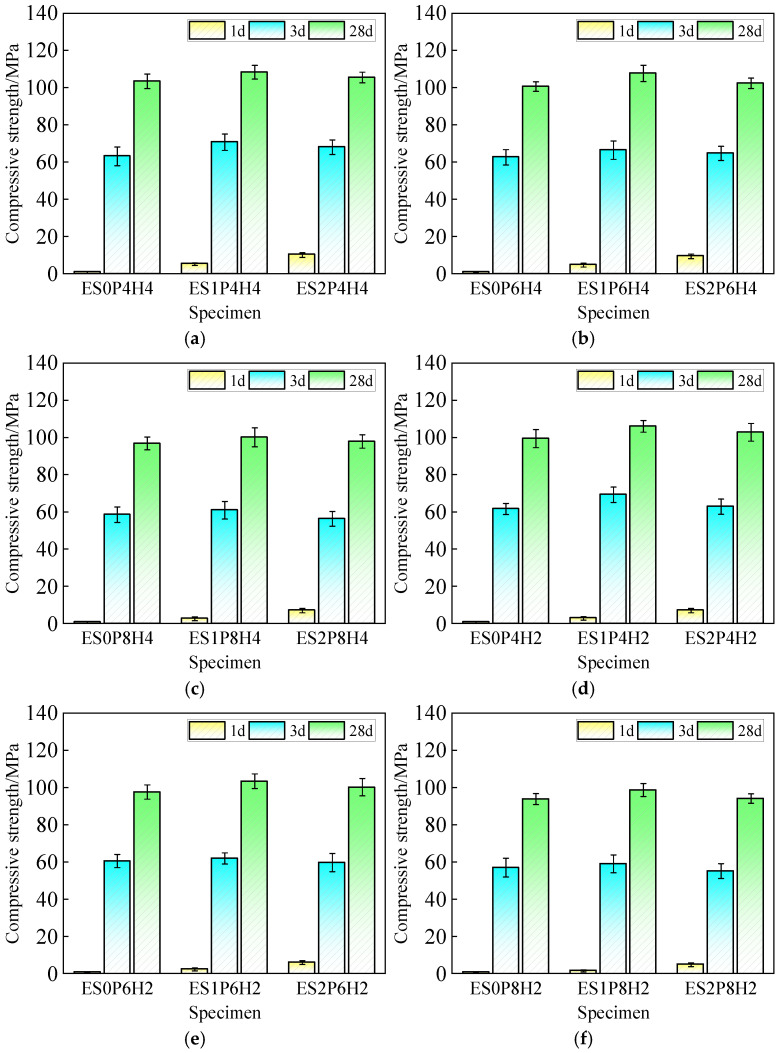
The effect of different dosages of “ES + PEA + HP-CSA” on the compressive strength of coral sand grouting materials: (**a**) PEA: 0.04%, HP-CSA: 4%; (**b**) PEA: 0.06%, HP-CSA: 4%; (**c**) PEA: 0.08%, HP-CSA: 4%; (**d**) PEA: 0.04%, HP-CSA: 2%; (**e**) PEA: 0.06%, HP-CSA: 2%; (**f**) PEA: 0.08%, HP-CSA: 2%.

Figure 8 illustrates the effects of varying dosages of ES and composite expansive agents on the compressive strength of coral sand grouting materials. The compressive strength of coral sand grouting materials increases with curing age. Without ES incorporation, the high fluidity of coral sand grouting materials leads to severe retardation and slow development of strength. Increasing ES dosage significantly enhances the 1-day compressive strength, whereas the 28-day strength initially increases slightly before decreasing. Taking ES0P6H4, ES1P6H4, and ES2P6H4 as examples, adding 1% and 2% ES increases the 1-day strength by 400% and 870%, respectively, compared to the ES-free group. For the 28-day strength, ES1P6H4 shows a 7.1% increase relative to ES0P6H4, while ES2P6H4 exhibits a 4.9% reduction compared to ES1P6H4. This phenomenon arises because ES accelerates slurry hydration to improve early strength. However, excessive ES dosage results in excessively rapid hydration of the paste, with its hydration products accumulating disorderly within the matrix, impeding the late-age growth and development of C-S-H gels and adversely affecting the late-age strength development of the materials [49,50]. Consequently, a 1% ES dosage enables coral sand grouting materials to achieve both high early strength and optimal later-age strength. Increasing PEA dosage leads to a decrease in compressive strength across all curing ages. Relative to ES1P4H4, ES1P6H4 and ES1P8H4 exhibit strength reductions of 9.1% and 47.3% at 1 d, 6.1% and 13.8% at 3 d, and 0.1% and 7.6% at 28 d, respectively. Meanwhile, increasing HP-CSA dosage facilitates the strength development of grouting materials. Relative to ES1P6H4, ES1P6H2 exhibits a compressive strength decrease of 48.0%, 6.8%, and 4.0% at each respective curing age. Gas generation from PEA induces physical expansion in grouting materials, leading to increased porosity within the matrix at early ages. This significantly reduces compressive strength. However, at 28 d, the advancement of cement hydration and continuous formation of HP-CSA expansive products effectively fill in the internal pores of the matrix, alleviating the adverse impact of PEA incorporation on grouting materials’ compressive strength [51]. An appropriate dosage of HP-CSA enhances the strength of coral sand grouting materials across all curing ages. A comprehensive analysis indicates that, within the scope of the study, the optimal ES dosage is 1%. This dosage enables coral sand grouting materials to achieve a sustained and stable vertical expansion rate within 48 h while satisfying the Class II fluidity requirements specified in the national standard.

### 3.3. The Development of the Shrinkage Deformation from 2 to 28 Days

**Figure 9:**
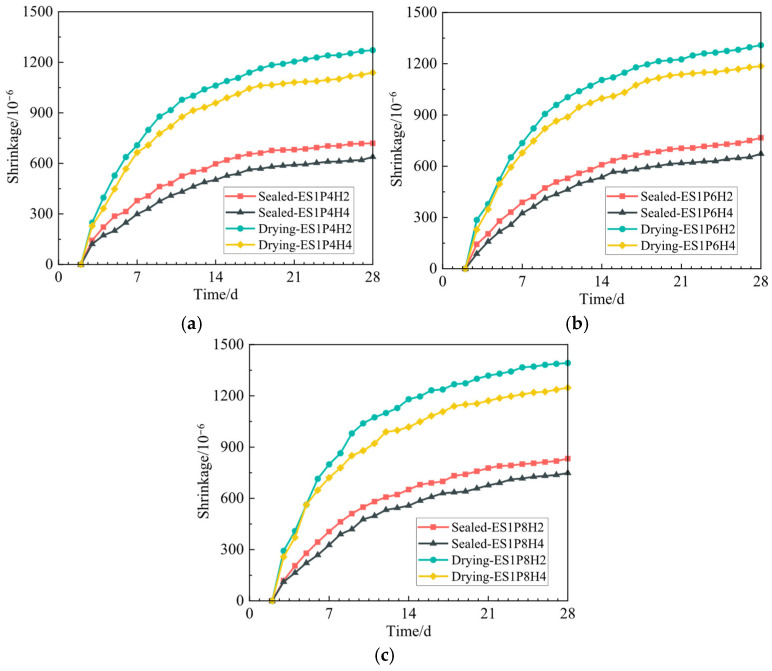
The effect of composite expansive agents on the shrinkage of coral sand grouting materials within 2–28 days: (**a**) PEA: 0.04%; (**b**) PEA: 0.06%; (**c**) PEA: 0.08%.

With the optimal ES dosage set at 1%, Figure 9 illustrates the influence of composite expansive agents on the autogenous shrinkage and drying shrinkage of coral sand grouting materials over 2–28 d. Shrinkage development under sealed curing corresponds to autogenous shrinkage, and development under drying conditions to drying shrinkage (it is the combined effect of autogenous shrinkage and net drying shrinkage). First, within this study, all coral sand grouting materials exhibit continuous shrinkage progression throughout 2–28 days. With the increase in curing age, the shrinkage of grouting materials gradually increases. The shrinkage develops rapidly at 2–7 d, the development rate gradually stabilizes at 7–14 d, and the rate further decreases by 28 d. The 14-day shrinkages reach 74.5–84.9% of the 28-day values, with 28-day shrinkage of all sealed specimens not exceeding 850 × 10^−6^ and that of drying-cured specimens not exceeding 1400 × 10^−6^.

Second, with increasing PEA dosage, the shrinkage effect of grouting materials increases correspondingly [31]. When the PEA dosage reaches 0.08%, the shrinkage effect is most pronounced. Under sealed conditions, the shrinkage of specimens in ES1P8H4 at 28 days is 748.25 × 10^−6^, which is 17.1% and 11.2% higher relative to the groups with 0.04% and 0.06% PEA dosage at the same curing age, respectively. Under drying conditions, the shrinkage of specimens in ES1P8H4 is 1247.51 × 10^−6^, increasing by 9.5% and 5.2% relative to the 0.04% and 0.06% PEA dosage groups at the same curing age, respectively. Excessive PEA increases the number of pores within the matrix, loosens the matrix structure, elevates shrinkage stress, reduces the material’s ability to resist shrinkage deformation, and ultimately causes grouting materials to exhibit significant shrinkage deformation.

Third, coral sand grouting materials exhibit smaller shrinkage deformation under sealed conditions relative to drying conditions. Taking the 28-day shrinkage as an example, at a fixed HP-CSA dosage of 4%, the autogenous shrinkage values of the groups with PEA dosages of 0.04%, 0.06%, and 0.08% are 638.75 × 10^−6^, 672.90 × 10^−6^, and 748.25 × 10^−6^, respectively, representing decreases of 43.9%, 43.2%, and 40.0% compared to the drying shrinkage values. Similarly, at a HP-CSA dosage of 2%, the corresponding autogenous shrinkage values are 719.65 × 10^−6^, 766.37 × 10^−6^, and 832.40 × 10^−6^, exhibiting reductions of 43.4%, 41.4%, and 40.2%, respectively. Cement continuously consumes water in capillary pores during hydration, generating capillary negative pressure within the matrix and thereby inducing autogenous shrinkage of grouting materials, whereas drying shrinkage primarily arises from a humidity gradient between the external environment and the interior of grouting materials, which drives the diffusion and loss of internal moisture [52]. Sealed conditions better inhibit moisture loss from grouting materials, providing free water for hydration reactions to offset part of the shrinkage effect. Additionally, as natural lightweight aggregates, coral aggregates have high water absorption [8,53]. During mixing, coral aggregates can absorb part of the water and provide an internal curing environment for subsequent hydration [9]. Under sealed conditions, the water stored in coral aggregates can be utilized to the maximum extent for internal matrix hydration, further suppressing early-age shrinkage of the material [54].

Fourth, within the same dosage of PEA, increasing HP-CSA dosage reduces both autogenous shrinkage and drying shrinkage of grouting materials over 2–28 d. ES1P4H4 exhibits 11.2% and 10.5% lower autogenous shrinkage and drying shrinkage, respectively, compared to ES1P4H2. At PEA dosages of 0.06% and 0.08%, these decreases are 12.2% and 9.4%, and 10.1% and 10.4%, respectively. The expansive products of HP-CSA can effectively fill internal matrix pores, improve their compactness, and compensate to some extent for the deterioration of shrinkage performance induced by PEA incorporation. However, the PEA dosage should not be excessively high. Overall, the combination of PEA and HP-CSA at appropriate dosages can effectively synergistically regulate vertical expansion and the development of shrinkage deformation in coral sand grouting materials over 0–48 h and 2–28 d, thereby exerting a relay and complementary effect.

Finally, at 28 d, within the same series, the shrinkage differences between drying specimens with 4% and 2% HP-CSA are all greater than those of the corresponding sealed specimens. For example, in the ES1P6H4 and ES1P6H2 groups, the drying shrinkage difference between them is 123.32 × 10^−6^, and the autogenous shrinkage difference is 93.47 × 10^−6^. This indicates that sealed curing is more favorable for HP-CSA to exert its shrinkage compensation effect on grouting materials.

**Figure 10:**
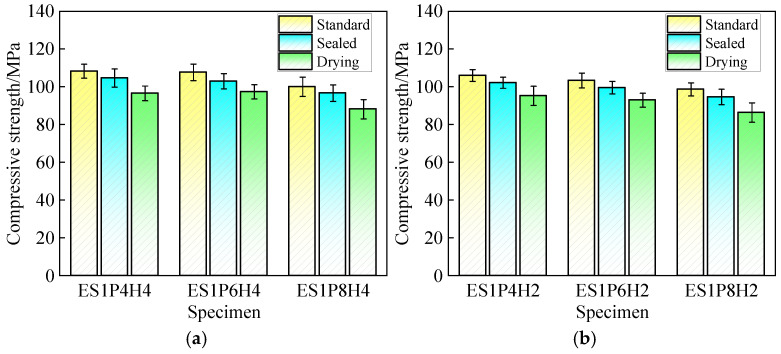
The 28-day compressive strength of coral sand grouting materials with different curing conditions: (**a**) HP-CSA: 4%; (**b**) HP-CSA: 2%.

Meanwhile, the study further investigates the variation pattern of 28-day compressive strength of specimens under three different curing conditions, as shown in Figure 10. Results indicate that compressive strength under standard curing is the highest, followed by sealed curing, with dry curing yielding the lowest values [55]. Taking ES1P6H4 as an example, compared with dry curing, the 28-day compressive strength of specimens under standard and sealed curing increases by 10.6% and 5.7%, respectively. Under sealed curing, as the available water within the matrix is limited, residual water in the system can hardly sustain the water demand of the hydration process in the later hydration stage, slowing the hydration rate and restricting strength development of grouting materials. Therefore, their strength is lower than that of specimens under standard curing. Comparative analysis reveals that the variation pattern of compressive strength across all groups aligns with the shrinkage development trend: when the curing environment and expansive agents’ dosage intensify the shrinkage effect, strength development is inhibited. Conversely, it promotes strength development.

### 3.4. The Drying Shrinkage and Water Loss Rate

**Figure 11:**
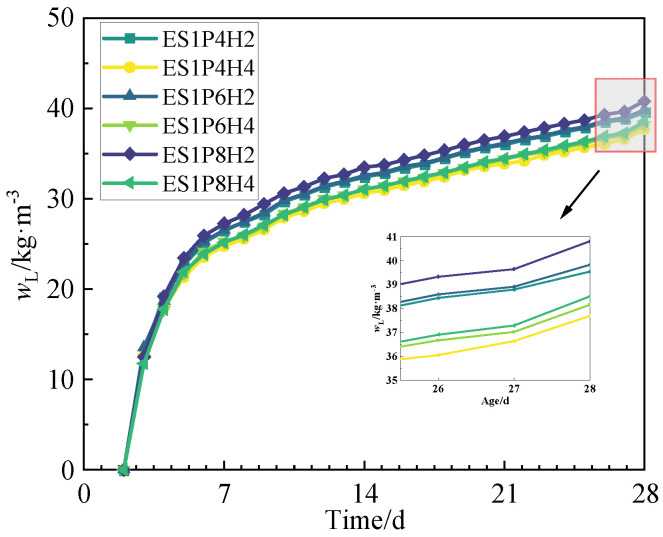
The development of the drying water loss rate within 2–28 d of coral sand grouting materials.

**Figure 12:**
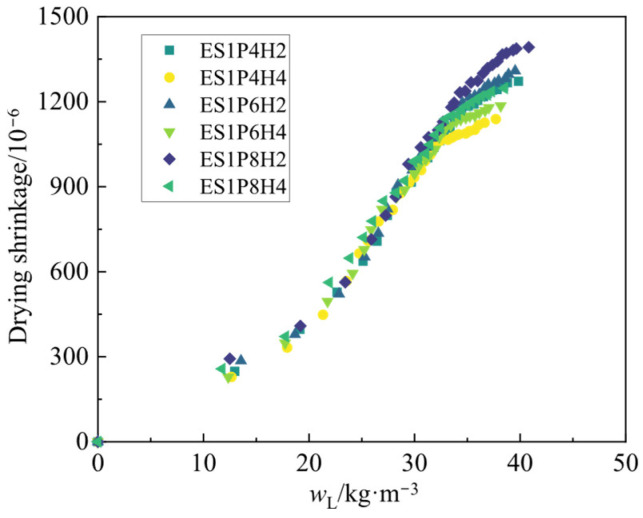
The relationship between the drying shrinkage and water loss rate of coral sand grouting materials.

To investigate the relationship between drying shrinkage and water loss rate of coral sand grouting materials, this study plots the drying water loss rate development curves for each mix proportion over the 2–28 day curing period, as shown in Figure 11. Analysis of the curves reveals that the drying water loss rate development trend across all groups is generally consistent with their drying shrinkage in that both gradually increase with extended curing age, albeit at a decelerating rate. At the 28-day curing age, the drying water loss rate of ES1P8H4 reaches 38.52 kg/m^3^, exhibiting increases of 2.2% and 0.9% relative to the groups with 0.04% and 0.06% PEA dosages, respectively. The drying water loss rate of ES1P6H2 is 3.6% higher than that of ES1P6H4. All these results follow the aforementioned trend. This aligns with the findings of Xia et al. [56] and Wang et al. [57]. Therefore, the drying shrinkage and drying water loss rate for each group are plotted in a correlation graph, as shown in Figure 12.

Analysis reveals that despite variations in PEA and HP-CSA dosages across different specimens, their drying shrinkage and drying water loss rates exhibit a distinct linear correlation [41]. The fitting results are presented in Table 5, with all determination coefficients (*R*^2^) exceeding 0.95, demonstrating high fitting accuracy. This indicates that the drying water loss rate of grouting materials can indirectly reflect its drying shrinkage behavior. Polar functional groups on the surface of hydration products (e.g., C-S-H gels) in grouting materials can form hydrogen bonds with internal water molecules, constraining the movement of water molecules [58]. As the curing age increases, greater quantities of hydration products form, enabling more binding of residual water molecules within the matrix. Stronger hydrogen bonds enhance the capacity to bind water molecules and slow water loss from the matrix. Macroscopically, this manifests as the gradual flattening of both drying shrinkage and water loss rate with increasing age. Within the scope of this study, the incorporation of composite expansive agents fails to fully offset the shrinkage effect over the 2–28 d period. However, analysis of the slopes from the fitting results shows that higher PEA and lower HP-CSA dosages result in steeper slopes. This provides indirect evidence for the adverse impact of excessive PEA on grouting materials’ shrinkage and the mitigative effect of an appropriate HP-CSA dosage on shrinkage.

In summary, based on the established mix proportions within this study, the coral sand grouting materials incorporating 1% ES, 0.06% PEA, and 4% HP-CSA demonstrates compliance with the national standard for Class II fluidity, attains a standard-cured compressive strength of 107.8 MPa, meets the specified vertical expansion rate criteria, and maintains shrinkage deformation within acceptable limits over 2–28 d.

### 3.5. Microstructure

**Figure 13:**
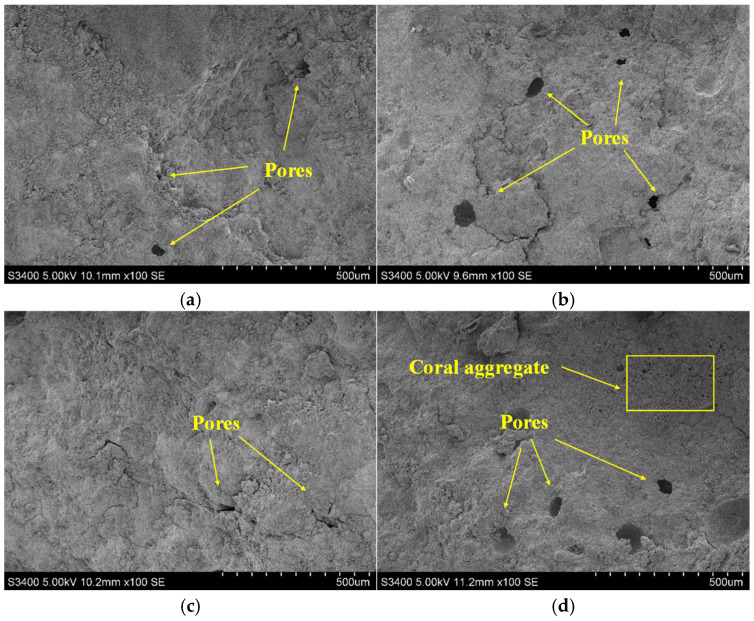
Microstructure of coral sand grouting materials at 1 day: (**a**) ES0P6H4; (**b**) ES1P6H4; (**c**) ES1P4H4; (**d**) ES1P6H2.

**Figure 14:**
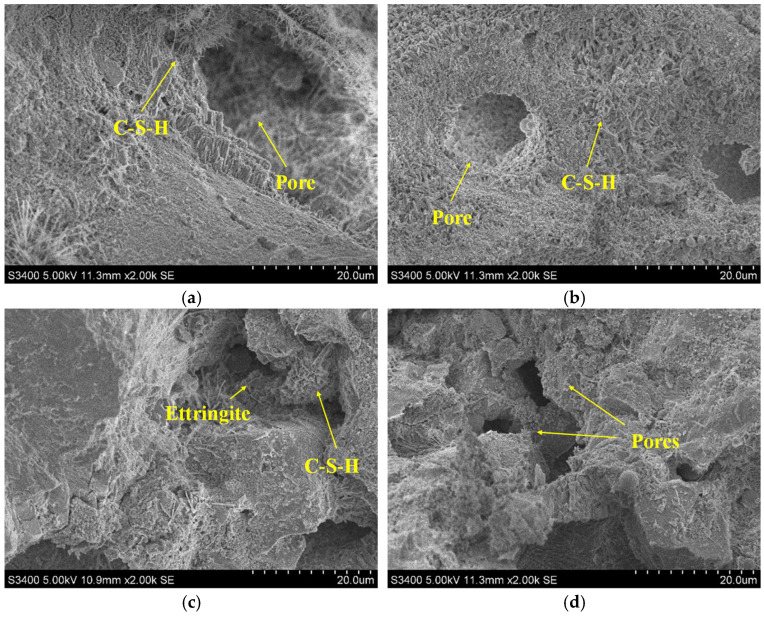
Microstructure of coral sand grouting materials at 28 days: (**a**) ES0P6H4; (**b**) ES1P6H4; (**c**) ES1P4H4; (**d**) ES1P6H2.

To verify the above conclusions, this study examines four groups (ES0P6H4, ES1P6H4, ES1P4H4, and ES1P6H2) to analyze their microstructural changes at 1 d and 28 days, respectively, as shown in Figure 13 and Figure 14. As the curing age increases, hydration products within and around the pores of coral sand grouting materials gradually accumulate and become denser. At 1 d, the internal structure of the material is relatively loose. In ES1P6H4, the incorporation of ES effectively inhibits the escape of gas generated by PEA, resulting in more pores than ES0P6H4. Additionally, with the increased PEA content, ES1P4H4 exhibits fewer pores than ES1P6H4 with a relatively stable structure. At 28 d, the continuous formation of hydration products renders the matrix structure more compact and stable. Compared to ES1P6H2, the internal pores of ES1P6H4 are partially filled, and the increased HP-CSA content compensates for internal pores, effectively mitigating the adverse effects induced by PEA incorporation.

## 4. Conclusions

This study investigated the effects of varying dosages of ES, PEA, and HP-CSA on the 0–48 h vertical expansion rate curve of coral sand grouting materials and analyzed the influence of composite expansion agents on 2–28 d shrinkage deformation development under different curing conditions. The main conclusions are as follows:

1. The combined regulation of ES, PEA, and HP-CSA enables effective synchronization between the expansion performance window of PEA and the early-age strength development of the grout, thereby realizing precise control over the 48–hour vertical expansion curve of coral sand grouting materials. Simply increasing the HP-CSA dosage accelerates the development of the vertical expansion curve, reduces the initial peak values, and shortens the time required to reach this peak. Within the scope of this study, the incorporation of appropriate dosages of calcium formate and HP-CSA can achieve secondary growth of vertical expansion rate in high-fluidity coral sand grouting materials during the ultra-early ages.
2. From 2 to 28 days, coral sand grouting materials exhibit continuous shrinkage development with gradually decreasing shrinkage rate, progressively approaching an equilibrium state. Dry curing condition exerts a significant influence on the shrinkage deformation of the material. An excessive PEA dosage induces substantial shrinkage deformation in coral sand grouting materials. The combination of appropriate dosages of PEA and HP-CSA can effectively and synergistically regulate the deformation development of coral sand grouting materials during both 0–48 h and 2–28 d, thereby enabling a staged and controllable design of its deformation evolution.
3. The drying shrinkage of coral sand grouting materials exhibits a positive correlation with the drying water loss rate, with all groups yielding *R*^2^ values exceeding 0.95, indicative of excellent fitting results. Different curing conditions induce significant variations in the 28-day compressive strength of coral sand grouting materials. Specimens subjected to standard curing achieved the highest strength, followed by those under sealed curing, whereas dry-cured specimens exhibited the lowest values.
4. Based on the established mix proportions within this study, a high-strength coral sand grouting material is developed by incorporating ES, PEA, and HP-CSA at dosages of 1%, 0.06%, and 4%, respectively. The material meets the Class II fluidity requirements specified in the national standard, complies with the criteria for vertical expansion rate within 24 h, and exhibits shrinkage deformation within a reasonable range from 2 to 28 days, with its 28-day compressive strength reaching 107.8 MPa.

Additionally, this study focuses solely on investigating the shrinkage deformation development of coral sand grouting materials with composite expansion agents within 0–28 d, while subsequent research can emphasize longer-term testing.

## Figures and Tables

**Table 1:** Performance test reference standards.

Test Performance	Standards	
Fluidity (mm)	Initial value	≥340
	30 min value	≥310
Vertical expansion rate (%)	3 h	0.10–3.50
	The difference between 24 h and 3 h	0.02–0.50

**Table 2:** Mineral composition of HP-CSA.

Component	CaO	CaSO_4_	Ca_4_Al_6_O_12_SO_4_	Ca(OH)_2_	CaCO_3_
Mass fraction (%)	47	36	7	7	3

**Table 3:** Mix proportions of coral sand grouting materials.

Component	Cement	FFA	Coral Sand	Water	PS	DA	RPP
Proportion (kg/m^3^)	715	306	1021	245	10	1.53	1.02

**Table 4:** Mix design for regulation of ES and composite expansive agents on early-age expansive properties of coral sand grouting materials.

Specimens	Cement (kg/m^3^)	HP-CSA (kg/m^3^)	PEA (kg/m^3^)	ES (kg/m^3^)
ES0P4H2	694.50	20.50	0.41	0
ES1P4H2	694.50	20.50	0.41	10.21
ES2P4H2	694.50	20.50	0.41	20.42
ES0P6H2	694.50	20.50	0.61	0
ES1P6H2	694.50	20.50	0.61	10.21
ES2P6H2	694.50	20.50	0.61	20.42
ES0P8H2	694.50	20.50	0.82	0
ES1P8H2	694.50	20.50	0.82	10.21
ES2P8H2	694.50	20.50	0.82	20.42
ES0P4H4	674.00	41.00	0.41	0
ES1P4H4	674.00	41.00	0.41	10.21
ES2P4H4	674.00	41.00	0.41	20.42
ES0P6H4	674.00	41.00	0.61	0
ES1P6H4	674.00	41.00	0.61	10.21
ES2P6H4	674.00	41.00	0.61	20.42
ES0P8H4	674.00	41.00	0.82	0
ES1P8H4	674.00	41.00	0.82	10.21
ES2P8H4	674.00	41.00	0.82	20.42

**Table 5:** The fitting results of the drying shrinkage and water loss rate of coral sand grouting materials.

Specimens	Fitting Equations	*R* ^2^
ES1P4H2	*y* = 37.86*x* − 193.14	0.9591
ES1P6H2	*y* = 38.82*x* − 199.67	0.9562
ES1P8H2	*y* = 40.13*x* − 200.09	0.9593
ES1P4H4	*y* = 36.17*x* − 177.19	0.9529
ES1P6H4	*y* = 37.14*x* − 181.73	0.9577
ES1P8H4	*y* = 37.74*x* − 162.40	0.9684

## Data Availability

The original contributions presented in the study are included in the article, further inquiries can be directed to the corresponding authors.
